# Distinct *Campylobacter fetus* lineages adapted as livestock pathogens and human pathobionts in the intestinal microbiota

**DOI:** 10.1038/s41467-017-01449-9

**Published:** 2017-11-08

**Authors:** Gregorio Iraola, Samuel C. Forster, Nitin Kumar, Philippe Lehours, Sadjia Bekal, Francisco J. García-Peña, Fernando Paolicchi, Claudia Morsella, Helmut Hotzel, Po-Ren Hsueh, Ana Vidal, Simon Lévesque, Wataru Yamazaki, Claudia Balzan, Agueda Vargas, Alessandra Piccirillo, Bonnie Chaban, Janet E. Hill, Laura Betancor, Luis Collado, Isabelle Truyers, Anne C. Midwinter, Hatice T. Dagi, Francis Mégraud, Lucía Calleros, Ruben Pérez, Hugo Naya, Trevor D. Lawley

**Affiliations:** 1 Unidad de Bioinformática, Institut Pasteur Montevideo, 11400 Montevideo, Uruguay; 2 Sección Genética Evolutiva, Facultad de Ciencias, Universidad de la República, 11400 Montevideo, Uruguay; 3 Host-Microbiota Interactions Laboratory, Wellcome Trust Sanger Institute, CB10 1SA Hinxton, UK; 4 Centre for Innate Immunity and Infectious Diseases, Hudson Institute of Medical Research, Clayton, VIC 3168 Australia; 5 Department of Molecular and Translational Sciences, Monash University, Clayton, VIC 3168 Australia; 6 Bordeaux Research in Translational Oncology, INSERM UMR1053, University of Bordeaux, 33076 Bordeaux, France; 7 French National Reference Center for Campylobacters and Helicobacters, University of Bordeaux, 33076 Bordeaux, France; 8 Laboratoire de Santé Publique du Québec, Institut National de Santé Publique du Québec, Sainte-Anne-de-Bellevue, QC Canada H9X 3Y3; 9 Départment de Microbiologie, Immunologie et Infectiologie, Université de Montréal, Montreal, QC Canada H3T 1J4; 10 Departamento de Bacteriología, Laboratorio Central de Veterinaria de Algete (MAGRAMA), 28110 Algete, Spain; 11 Laboratorio de Bacteriología, EEA-INTA Balcarce, Balcarce, 7620 Argentina; 12 Friedrich-Loeffler-Institut, Institute of Bacterial Infections and Zoonoses, 07743 Jena, Germany; 13 Departments of Laboratory Medicine and Internal Medicine, National Taiwan University Hospital, Taipei, 10617 Taiwan; 14 Animal and Plant Health Association (APHA), Addlestone, KT15 3NB UK; 15 Department of Veterinary Science, Faculty of Agriculture, University of Miyazaki, Miyazaki, 889-2192 Japan; 16 Departamento de Medicina Veterinária Preventiva, Universidade Federal de Santa Maria, Santa Maria, 97105-900 Brazil; 17 Department of Comparative Biomedicine and Food Science, University of Padova, Padova, 35122 Italy; 18 Faculty of Science, Health, Education and Engineering, University of the Sunshine Coast, Sippy Downs, QLD 4556 Australia; 19 Department of Veterinary Microbiology, University of Saskatchewan, Saskatchewan, SK Canada S7N 5A2; 20 Instituto de Higiene, Facultad de Medicina, Universidad de la República, Montevideo, 11600 Uruguay; 21 Instituto de Bioquímica y Microbiología, Facultad de Ciencias, Universidad Austral de Chile, 5090000 Valdivia, Chile; 22 Royal (Dick) School of Veterinary Studies, University of Edinburgh, Midlothian, EH25 9RG UK; 23 EpiLab, Infectious Disease Research Centre, Massey University, Palmerston North, 4442 New Zealand; 24 Department of Microbiology, Faculty of Medicine, Selçuk University, Selçuklu, 42250 Turkey; 25 Departamento de Producción Animal y Pasturas, Facultad de Agronomía, Universidad de la República, 12900 Montevideo, Uruguay

## Abstract

*Campylobacter fetus* is a venereal pathogen of cattle and sheep, and an opportunistic human pathogen. It is often assumed that *C. fetus* infection occurs in humans as a zoonosis through food chain transmission. Here we show that mammalian *C. fetus* consists of distinct evolutionary lineages, primarily associated with either human or bovine hosts. We use whole-genome phylogenetics on 182 strains from 17 countries to provide evidence that *C. fetus* may have originated in humans around 10,500 years ago and may have “jumped” into cattle during the livestock domestication period. We detect *C. fetus* genomes in 8% of healthy human fecal metagenomes, where the human-associated lineages are the dominant type (78%). Thus, our work suggests that *C. fetus* is an unappreciated human intestinal pathobiont likely spread by human to human transmission. This genome-based evolutionary framework will facilitate *C. fetus* epidemiology research and the development of improved molecular diagnostics and prevention schemes for this neglected pathogen.

## Abstract (web summary)

Human infections with *Campylobacter fetus* are often assumed to be derived from livestock. Here, Iraola et al. provide evidence that healthy humans may act as carriers and dispersers, and *C. fetus* may have originated in humans as an intestinal pathobiont and then adapted as a livestock pathogen.

## Introduction

The species *Campylobacter fetus* is currently divided in three subspecies based on traditional biochemical and genotyping methods: *C*. *fetus* subsp. *venerealis* (Cfv) and *C. fetus* subsp. *fetus* (Cff) are associated with infections in mammals^1–3^, while *C. fetus* subsp. *testudinum* (Cft) is primarily isolated from reptiles^4^. The application of multilocus sequence typing (MLST) has shown that reptile-associated Cft are genetically distant from mammal-associated Cfv and Cff, which show high genetic relatedness^5, 6^. More recently the comparison of whole-genome sequences confirmed that Cft and Cff/Cfv represent distinct and divergent evolutionary lineages associated with reptiles and mammals, respectively^7^. Despite the importance of Cff/Cfv in livestock and human health, its genomic evolution within mammals remains poorly understood. Two recent whole genome based studies revealed incongruences between the phylogenetic structure of the Cff/Cfv population and the biochemical features used for their discrimination, questioning the clinical relevance of subtyping mammal strains^8, 9^. Accordingly, effective infection prevention and control schemes require a robust phylogenetic framework describing the host-associated evolution of *C. fetus* in mammals. Many important questions remain unanswered regarding the evolutionary relationship between strains isolated from bovine and human hosts, the transmission patterns of *C. fetus* between mammal hosts, and the actual potential of this species as a zoonotic pathogen.

In this work, we evidence the presence of distinct *C. fetus* lineages that have primarily adapted to humans or cattle. We propose that *C. fetus* may have originated as a human pathobiont present in the intestinal microbiota of healthy individuals and then host jumped to cattle and adapted as a venereal pathogen. Our work provides the phylogenetic and evolutionary framework to guide the development of methods for differentiation and epidemiological surveillance of the bovine and human lineages.

## Results

### A global *C. fetus* collection

To investigate the population structure and genomic evolution of mammal associated *C. fetus* we whole-genome sequenced 177 *C. fetus* strains isolated from 13 different countries and 5 different hosts, and combined our data with 11 published genomes sampled from 4 additional countries, giving a total data set of 188 genomes coming from strains isolated from 1952 to 2015 (63 years). To confirm species membership, the average nucleotide identity (ANI) was calculated for all possible pairs of genomes^10^. This analysis revealed a group of 6 genomes with an ANI <95% compared to the other genomes (two public and four sequenced as part of our study), which were assigned to the subspecies Cft due to high genomic divergence and were therefore removed from subsequent analysis. It is noteworthy that Cff/Cfv and Cft could be classified in two distinct species (ANI<95%). The remaining 182 Cff/Cfv genomes belong to a single species (ANI>95%; Supplementary Fig. 1) and were isolated from 4 different mammal hosts: 91 from bovines, 77 from humans, 13 from ovines and 1 from a monkey in captivity (Supplementary Data 1 and https://microreact.org/project/Bke4QRtHx).

### Phylogenetic structure, host-association and transmission

**Fig. 1:**
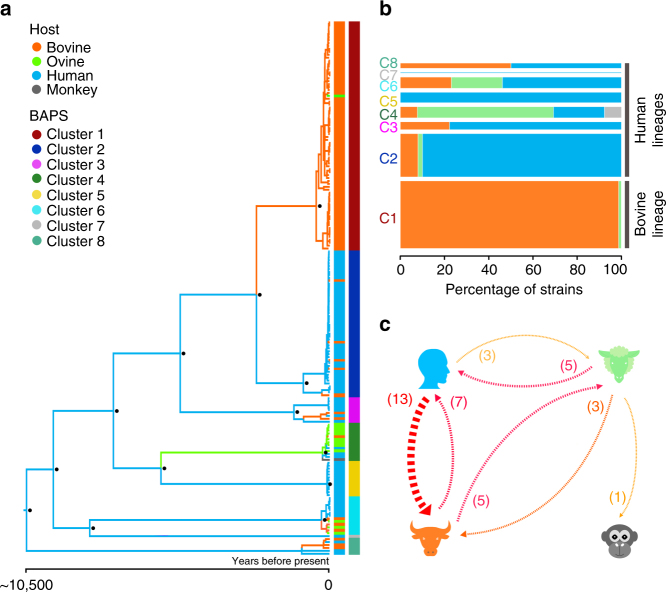
Phylogenetic structure and transmission. **a** Time-scaled phylogeny of the *C. fetus* population constructed from the core genome alignment (980 genes). Branches are colored according the most probable ancestral host. Vertical color strips are colored according to host type and to BAPS clustering, respectively. **b** Bar plot showing the distribution of hosts in each BAPS cluster, bar width is proportional to the percentage of strains present at each cluster. **c** Asymmetric graph showing significant host-to-host transmissions. Arrows redness is proportional to the transmission rate and the width is proportional to the Bayes factor

We built a time-scaled phylogeny and applied a Bayesian-clustering method (BAPS)^11^ over the core genome to understand the genetic structure of the *C. fetus* population (Supplementary Fig. 2). BAPS identified eight clusters consistent with the observed phylogenetic structure (Fig. 1a). The average number of core genome SNPs for isolates belonging to the same cluster was 90 (IQR = 38–142) while it increased to 558 for isolates belonging to different clusters (IQR = 231–885). Based on the phylogeny we estimated the time of divergence from the most recent common ancestor for the eight clusters to ~10,500 years ago (95% HPD = 8000–14,000). This estimation was not affected by confounding effects caused by previously identified sampling biases^12–14^ (Supplementary Figs. 3, 4; Methods section). The average substitution rate was 2.9 × 10^−5^ s/s/y (95% HPD: 2.1 × 10^−5^–5.1 × 10^−6^), which is comparable to estimations made for *C. jejuni* (around 3.2 × 10^−5^ s/s/y)^15^ and falls within the interval inferred for different gram-negative and gram-positive bacterial data sets with temporal structure^16^. Comparison of the genetic structure defined by the phylogeny and the host type of each isolate using a Bayesian Tip-association Significance (BaTS) test^17^ revealed a significant association of the population structure to each host type, particularly for human and bovine hosts (Supplementary Fig. 5). The uneven distribution of host type across the different *C. fetus* clusters is shown Fig. 1b. As a general trend, cluster 1 is associated to bovine hosts (with 99% of bovine strains) and is referred to as the modern bovine lineage. The origin of this lineage was traced around 2500 years ago (95% HPD = 1500–4000), suggesting the establishment of a successful genotype adapted to bovine hosts. Clusters 2 to 8 are mainly associated to non-bovine hosts, with up to 100% of human strains, hence are hereinafter referred as the human lineages. Thus, our results are consistent with a strong host-associated evolution of *C. fetus* lineages that began around 10,500 years ago (95% HPD = 8000–14,000). Interestingly, this corresponds to the time when humans began to domesticate cattle^18^.

To infer the host-to-host transmission patterns of *C. fetus* we reconstructed the ancestral states for the internal nodes of the phylogeny and quantified each specific type of host transmission. An asymmetric transition model was found to be more consistent with the data than a symmetric model (Bayes factor = 31), and a median of 37 host jumps were inferred along the evolutionary history of *C*. *fetus*. By far the most significant directional jump was from human to bovine hosts (Bayes factor = 124), however the reverse transmission from bovines to humans was also supported (Fig. 1c). For most ancestral nodes, including the *C. fetus* MRCA, the posterior probability for a human ancestral host was consistently higher (~0.5) than for the other hosts (Supplementary Figs. 6, 7; Methods section) supporting a human origin of the currently sampled mammal *C. fetus* population. This was also suggested by the identification of significant switches in the distribution of bovine hosts across the *C. fetus* phylogeny (Supplementary Fig. 8) and by the higher nucleotide sequence diversity (Π) observed for human isolates in comparison with bovine isolates (Supplementary Fig. 9). Our results support that humans were the original mammalian host of the currently sampled *C. fetus* population and provide a potential connection between cattle domestication and the modern bovine lineage that subsequently evolved within these hosts.

### Adaptive selective pressures

**Fig. 2:**
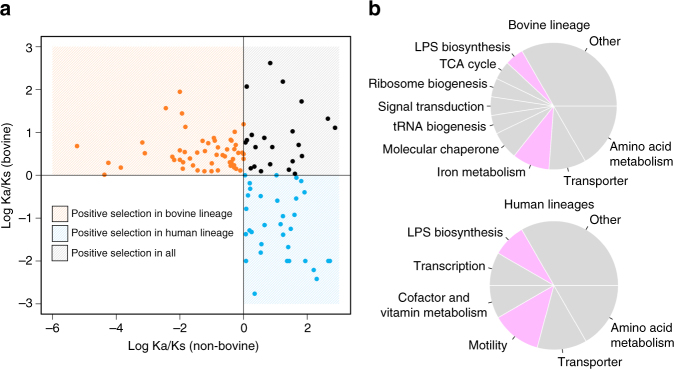
Positive selection in the core genome. **a** The scatter plot shows genes with Ka/Ks>1 in the bovine or human lineages. For better visualization the values are showed as the logarithm of the Ka/Ks ratio. **b** The pie charts show the distribution of biological process assignments to the genes with positive selection exclusively in bovine and human lineages. Categories involved in bacterial virulence are highlighted in violet. Categories with at least two genes were plotted

To investigate the adaptive evolution of the bovine and human lineages of *C. fetus* we analyzed the core genes under positive selection (Fig. 2a and Supplementary Table 2). A total of 32 genes were positively selected regardless of the host. A set of 33 genes were under positive selection exclusively in human lineages and the strongest signal was observed for the flagellar hook cap protein FlgD. The presence of diversifying alleles of flagellar genes, including *flgD*, has been found as a defining feature of hyper-invasive *C. jejuni* strains^19^. This may suggest parallels with the invasive and systemic bacteremia observed in many human-associated *C. fetus* infections. A distinct set of 30 genes were positively selected only in the bovine lineage and the enterobactin uptake receptor *cfrA* presented the strongest signal. The expression of the CfrA protein is induced under iron-restricted conditions and plays a crucial role in iron scavenging and in vivo colonization of other species like *C. jejuni* ^20^. Thus, the core genomes of the bovine and human lineages of *C. fetus* contain signatures of diversifying evolution as they are adapted in a unique way to interact with their hosts.

### A host-adapted accessory genome

To further explore the host-association of the *C. fetus* populations, we defined the accessory genomes and observed signals of adaptive evolution. First, we evidenced the presence of recombinant blocks shared by different phylogenetic clusters that are unlikely to be explained by the clonal frame (Supplementary Fig. 10a). Second, we explored the relationship between the synonymous divergence in the core genome and the genomic fluidity of the accessory genome to demonstrate the absence of a significant correlation between these two measures (Supplementary Fig. 10b), hence supporting adaptive evolution. Third, we found a positive and significant correlation between the genomic fluidity and the number of positions within recombinations (Supplementary Fig. 10c). Finally, we found substantial signals of homoplasy in more than 50% of the accessory genes using a maximum-parsimony approach (Supplementary Fig. 11a, b; Methods section). These findings led us to conclude that *C. fetus* accessory gene patterns are substantially influenced by an adaptive rather than neutral evolutionary forces.

**Fig. 3:**
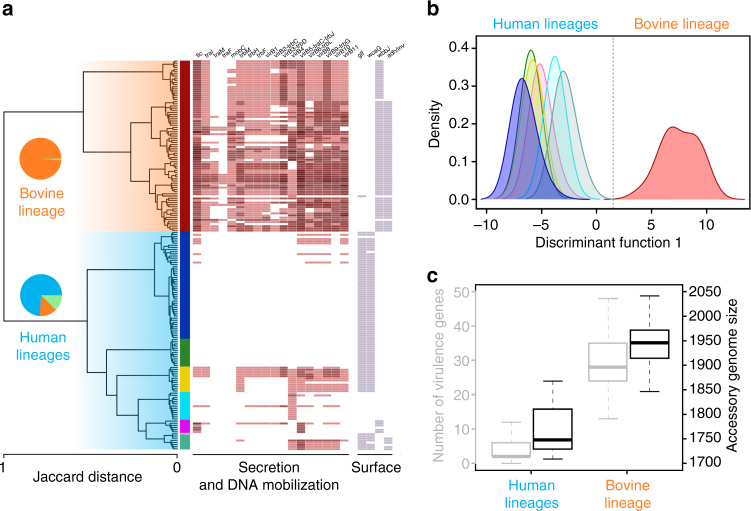
Accessory genome analysis. **a** Hierarchical clustering based on pairwise Jaccard distances, the distribution of hosts in the two main groups is presented as pie charts. The vertical color strip shows the correspondence of the accessory genome groups with the BAPS clusters identified with the core genome. The presence of virulence genes is shown as heatmap blocks whose opacity is proportional to the copy number. **b** Discriminant analysis of principal components (DAPC) showing the complete separation between bovine and human lineages based on the accessory genome. **c** Boxplots showing the abundance of virulence genes (gray) and all the accessory genes (black) in bovine and human lineages

Figure 3a shows how accessory gene patterns are differentially distributed across host-adapted lineages. Also we revealed that the accessory genome of the bovine lineage is substantially larger than that of human lineages, and contains a unique set of accessory genes including those putatively involved in host–pathogen interactions (Fig. 3b, c). For example, conjugative transfer (*tra*/*trb*) systems and type IV secretion system (T4SS) genes (*virB1*–*virB11*) were more frequent in the bovine lineage (Supplementary Fig. 12). T4SS genes have been found previously in *C. fetus* carried on plasmids or pathogenicity islands^21, 22^, and here we evidence they were potentially horizontally acquired from other *Campylobacter* and *Helicobacter* species (Supplementary Table 3 and Supplementary Fig. 13). T4SS are used to transfer plasmids between bacteria or to deliver virulence effectors to host cells in a variety of pathogens such as *Helicobacter pylori* ^23^. We also noted differences between the lineages in the LPS biosynthesis pathway genes which has important roles in LPS structures and host-interactions. For example, the *glf* (UDP-glucopyranose mutase) and *wcaG* (GDP-fucose biosynthesis) genes were more abundant in human lineages while *wbbJ* (galactoside O-acetyltransferase) was more frequent in the bovine lineage (Supplementary Fig. 12). Thus, the accessory genomes of bovine and human lineages harbor distinct gene sets that are potentially important for genome evolution and host–pathogen interactions, and likely reflect the adaptations to different hosts.

Interestingly, other accessory genes involved in blocking the incorporation of foreign DNA, like Cas protein coding genes, were more abundant in human lineages (Supplementary Fig. 14a). The CRISPR spacer loci were also unevenly distributed in bovine and human lineages (Supplementary Fig. 14b), supporting previous evidence of their suitability for host type tracking^24^. Restriction–modification system genes (R–M), which also play a role in maintaining genome integrity after foreign DNA incorporation^25^, also grouped genomes according to host type (Supplementary Fig. 15a). In particular, the abundance of type I R–M genes was greater in bovine respect to human lineages (Supplementary Fig. 15b). Hence, the differences in CRISPR/Cas and R–M systems may explain the smaller accessory genomes in human lineages and the stabilization of horizontally acquired DNA in the bovine lineage, contributing to host adaptation.

### A pathobiont in the intestinal microbiota

**Fig. 4:**
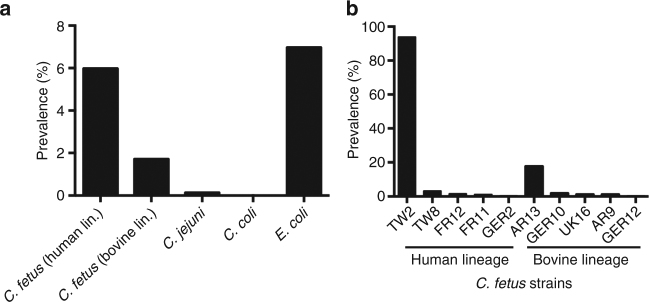
Human gut microbiome analysis. **a** Prevalence of *C. fetus* strains of human and bovine origin compared to prevalence of common pathogenic *Campylobacter* species (*C. jejuni* and *C. coli*) and *E. coli* in the gut microbiota of healthy humans. **b** Prevalence of *C. fetus* genotypes found in the intestinal microbiota of healthy individuals

Taken together, the genomic distinctions observed in the bovine and human lineages raise the possibility of having distinct natural reservoirs. Considering that other farm animals, like poultry and pigs, are not considered reservoir hosts^26, 27^ we hypothesized that *C. fetus* could be an unrecognized member of the human gastrointestinal microbiota. To determine if healthy humans may act as natural reservoir of *C. fetus*, we compared all genomes (*n* = 182) to the Human Pan-Microbe Communities database^28^. This allowed us to scan 7121 shotgun metagenomic data sets representing an international sampling of human feces. Interestingly, *C. fetus* was detected in ~8% of the samples from healthy humans. In contrast, the commensal *Escherichia coli* was detectable in 7% of the samples and the human pathogens *C. jejuni* and *C. coli* were detected in less than 0.5% of the samples. Of the 10 *C. fetus* genotypes detected in the gastrointestinal microbiota of healthy individuals, 22.11% belonged to strains from the modern bovine lineage and 77.89% belonged to strains from human lineages (Fig. 4a). No correlation was observed with gender, sex, age, ethnicity, or geographical origin of the donor or originating sample. Equally, co-abundance data showed no enrichment in other members of the microbiota. Taken together, this data suggests that microbiota community structure is not a determinant for *C. fetus* colonization success. Surprisingly, of the strains found from the human lineages, TW2 represented 94% of strains found within healthy individuals suggesting clonal expansion of this genotype in the sampled human population (Fig. 4b). The TW2 genotype belongs to cluster 5 which also includes the CA24 genotype that was responsible for a *C. fetus* outbreak among men who have sex with men, where human-to-human transmission was highly probable^29^.

## Discussion

Our results support a broad view of the natural history of *C. fetus* where the bacterium originally colonized humans, likely the gut microbiota. Then within the past 10,500 years *C. fetus* likely began to colonize and adapt to cattle, giving rise to a modern day bovine lineage that displays significant genomic distinctions from the other genomes belonging to the human lineages, linked to host-microbe interactions and genome stability. This observation is reminiscent to evolutionary patterns observed in other bacteria that moved between humans and animals^30, 31^. Furthermore, the presence of distinctive patterns of accessory genes in the bovine and human lineages is mostly explained by adaptive evolution. This is supported both by empirical evidence of homoplasy provided here and by recent theoretical models^32, 33^ that explain the emergence of these genomic distinctions as an adaptive process linked to niche transitions, such as the host jump observed in *C. fetus*. We also provide evidence that human-adapted *C. fetus* acts as an intestinal pathobiont capable of asymptomatic carriage and likely human to human transmission. Our work supports a previously unappreciated source of infection and transmission essential to properly understand the epidemiology of symptomatic *C. fetus* infections in humans. Interestingly, the absence of the bovine associated lineage in symptomatic individuals in our analysis suggests that zoonotic transmission of the bovine *C. fetus* lineage to humans is not as common as human-to-human transmission. In contrast, the detection of the human-associated lineages in bovine samples introduces humans as a possible source of infection to cattle. Together, these observations will guide the development of phylogenetically based genetic markers capable of differentiating bovine and human lineages, and inform *C. fetus* diagnosis and epidemiology.

## Methods

### Sequencing, species typing, and pan-genome

Strains were retrieved from independent collections over the world (information of each sequenced strain can be found in Supplementary Data 1). Genomic DNA was prepared and sequenced using the Illumina Hi-Seq platform with library fragment sizes of 200–300 bp and read length of 100 bp at the Wellcome Trust Sanger Institute, as previously described^34^. Each sequenced genome was de novo assembled and improved using an in-house pipeline developed at the Wellcome Trust Sanger Institute^35^. Briefly, raw reads are first screened with Kraken^36^ software in order to discard significant amounts of contaminating reads from other organisms. Data summary statistics are then generated using FastQC (www.bioinformatics.babraham.ac.uk/projects/fastqc). Then, reads are assembled using Velvet^37^ to generate multiple assemblies by varying the k-mer size between 66 and 90 % of the read length using with VelvetOptimiser^38^. From this set of assemblies the one with highest N50 is chosen. Iterative assembly improvement steps are subsequently run to scaffold the contigs using SSPACE^39^ and to fill in sequence gaps using GapFiller^40^. Finally, contigs are excluded from the assembly if they are shorter than the target fragment size (normally 300–500 bases). Remaining contigs are sorted by size and renamed in a standardized manner to include the raw sequencing data accession number. To assess the quality of the assembly and to produce summary statistics, the reads are aligned back to the final assembly using SMALT^41^. The assemblies are then automatically annotated using Prokka^42^ with a genus-specific database from RefSeq. The resulting annotated assemblies are in GFF3 format. A more detailed explanation of parameters used for each software at each step can be found in Page et al.^35^ The membership to the species *C. fetus* was assessed by comparing each assembled genome against each other and the published genomes using the ANI, as previously implemented^43^.

The annotated assemblies in GFF3 format were input to Roary, a tool that builds large-scale pan-genomes, to construct the core and accessory genomes^44^. Briefly, coding sequences are extracted from annotation files and converted into protein sequences, filtered to remove partial sequences and iteratively pre-clustered with CD-HIT^45^. Then all-against-all comparisons of pre-cluster representatives are performed with BLASTP using an user defined percentage sequence identity cut-off. Sequences are then clustered with MCL^46^ and finally the pre-clustering results from CD-HIT are merged together with the results of MCL. Then homologous groups containing paralogs are split into groups of true orthologs using conserved gene neighborhood information. A graph of relationships between clusters is constructed based on their order of occurrence in the input sequences for providing context to each gene. Full details of the method and outputs are provided in the original paper by Page et al.^44^ As the percentage sequence identity cut-off is an important parameter for defining core and accessory gene sets we ran multiple pan-genome estimations by varying this parameter from 80 to 95% by a step of 5%. Results for this analysis are shown in Supplementary Fig. 2. As no significant differences were found fundamentally in the size of the inferred core genome, all the analyses were performed with 90% of identify. Additionally, to check the core genome quality we inspected that the housekeeping genes belonging to the *C. fetus* MLST scheme clustered in the corresponding homologous groups.

### Phylodynamics, population structure, and host jump analyses

The core genome alignment extracted with Roary (1,098,169 aligned positions representing 980 concatenated single copy genes) was filtered using Gubbins^47^ to remove high SNP density regions which indicate putative recombination events (44,213 positions removed representing ~4% of the core genome alignment). We used the BEAST v1.7.5 package^48^ to jointly estimate the substitution rate, a dated phylogeny and the reconstruction of host ancestral states using a continuous-time Markov chain (CTMC) discrete model. Briefly, we tested various combinations for the molecular clock prior (strict vs. relaxed), the demographic function prior (constant, exponential, and logistic) and the host jump model (symmetric vs. asymmetric). We analyzed four independent runs using 100 million generations sampled every hundred steps, checking for effective sample sizes (greater than 200 for each parameter) to ensure convergence. A burn-in of 10% of states was discarded from each run. Models were compared by calculating Bayes factors (BFs) using the stepping stone algorithm^49^. Since no significant differences were found for the molecular clock and demographic function priors we used a relaxed-lognormal clock with a constant population function. A significant BF was found supporting an asymmetric over a symmetric CTMC discrete model. The significance of each directional host jump was assessed by calculating BFs over the non-zero rates. We also used the approach described in TreeBreaker^50^ for identifying branches in the *C. fetus* tree where the distribution of hosts has significantly changed. Sequence nucleotide diversity (Π) was calculated with the APE v4.0 package^51^. Phylogenetic trees were visualized using R v3.2.1^52^ and Microreact^53^.

To evaluate the population structure observed in the core genome phylogeny, we compared it with the output of HierBAPS^11^. This approach performs a Bayesian analysis of population structure to cluster similar samples based on their genomic relatedness. We used two clustering layers and 5, 10, 20, and 30 expected numbers of clusters (k) as input parameters. The monophyletic clades seen in the phylogeny totally agreed with the first layer of clustering. The association between the phylogenetic structure and host types was assessed with Befi-BaTS software v0.1.1^17^.

### Accounting for confounding effects for time-dating and sampling bias

To account for the confounding effects due to sampling biases of the *C. fetus* collection we implemented different approaches. First, we built two additional data sets subsampled from the complete data set, one just with European strains to minimize the uneven sampling at distant geographic regions, and other with the same number of strains belonging to each host type to minimize the overrepresentation of bovine and human strains at some phylogenetic clusters. Second, we built another two data sets by filtering out alignment sites likely to be subject to selection: one approach consisted in removing 106 core genes with evidence of negative selection (d*N*/d*S* << 1) and the 95 previously identified core genes with evidence of positive selection (d*N*/d*S*>1); giving a filtered core genome alignment representing 779 neutrally evolving genes (839,185 aligned positions). The other data set was generated by calculating position-specific d*N*/d*S* values across the core genome alignment using the “kaksCodon” function from the CorMut package^65^, this resulted in masking 9032 positions with evidence of negative (d*N*/d*S* << 1) or positive (d*N*/d*S* > 1) selection. Over all data sets we first evaluated the presence of time signal by calculating the linear regression between the root-to-tip distance and the isolation year. Null distributions of R^2^ values were generated by 1000 permutations on the actual isolation year using three approaches: (i) random permutations, (ii) clustered permutations as suggested by Murray et al.^54^, and (iii) clustered permutations by inputing the clusters according to BAPS results. Then, we implemented the Bayesian clustered permutation approach also described in Murray et al.^54^ using the “rand.xmls.R” R script to generate the randomized inputs to BEAST analyses. To account for confounding effects due to sampling bias we applied the CTMC host state reconstruction analysis in BEAST by performing 10 independent runs with the complete data set and both subsampled data sets (European and host-balanced). Posterior estimates for the bovine and human host states at the MRCA node are displayed as boxplots in Supplementary Fig. 7.

### Analysis of selective pressures

The aligned nucleotide sequences of each 980 single copy core genes were extracted from Roary’s output. The ratio between the number of non-synonymous mutations (Ka) and the number of synonymous mutations (Ks) was calculated for the whole alignment and for the respective subsets of strains belonging to the bovine and human lineages. The Ka/Ks ratio for each gene alignment was calculated with SeqinR v3.1. A Ka/Ks > 1 was considered as the threshold for identifying genes under positive selection. To evaluate the strength of the positive pressure acting over the same gene in bovine and non-bovine strains, we used the ranked absolute difference between the individual Ka/Ks ratios calculated over each subset of the corresponding alignments.

### Accessory genome analyses

The accessory genome was defined as gene sets present in less than 100% of the analyzed genomes. The Jaccard pair-wise distance between accessory gene patterns was calculated with the APE v4.0 package^51^. A discriminant analysis of principal components was used to identify differences in the distribution of accessory genes across the genomes, as implemented in the Adegenet v2.0.1 package^55^. The identification of virulence genes was performed by comparing the annotated protein sequences of each genome with Blast + blastp^56^ against the VFDB database^57^, the Victors database (http://www.phidias.us/victors/) and the PATRIC database of manually curated virulence genes^58^. First, genes were considered as present with query coverage or subject coverage ≥80% and identity ≥80%. Then, a second round of blastp was performed as previous but incorporating into the databases the *C. fetus* genes recovered from the first round, in order to minimize possible false negatives due to sequence divergence. The same approach was used to confirm the presence or absence of accessory genes present in bovine-associated or human-associated lineages. The Cas genes were identified by running hmmsearch^59^ (*e*-value < 1 × 10^−5^) against the Cas HMM profiles retrieved from Pfam^60^. The CRISPR spacers were identified with CRISPRdetect^61^.

### Evaluation of adaptive evolution

To evaluate the presence of adaptive signals governing the evolution of accessory genes we implemented two main approaches: (i) as described by Andreani et al.^62^, the correlation between synonym diversity (a measure of effective population size) in the core genome and genomic fluidity^63^ (a robust measure of accessory genome diversity) can be used to test for neutral evolution. We calculated genomic fluidity from accessory gene patterns as the ratio of unique gene families to the sum of gene families in pairs of genomes averaged over randomly chosen genome pairs from within a group of sampled genomes. For this we took 10,000 random samples of 50 genomes. Then we performed a linear regression analysis of genomic fluidity values against synonym diversity values calculated over the same random samples by applying natural logarithms to both measures. The same regression analysis was performed using the genomic fluidity values against the number of recombinant bases present on each sample. (ii) To test if the observed accessory gene patterns are not likely to be explained only by the clonal frame (evidence of homoplasy), for each accessory gene we calculated its consistency index (CI) using phangorn^64^. CI is defined as minimum number of changes divided by the number of changes required on the tree by maximum parsimony, and is equal to one if there is no homoplasy. We then set an empirical threshold of CI<0.25 to define genes with evidence of homoplasy.

### Analysis of human gut metagenomes

Human gastrointestinal metagenome analysis was performed using the Human Pan-Microbe Community database (http://www.hpmcd.org/). Genomes were included in the normalized database generation and scanned using the standard scanning algorithm. Abundance was scaled by genome uniqueness as described previously^28^. Abundance was scaled by read count for sample comparison.

### Data availability

The assembled genomic data and raw sequences have been deposited in the European Nucleotide Archive under the accession codes provided in Supplementary Data 1. The authors declare that all other data supporting the findings of the study are available in this article and its Supplementary Information files, or from the corresponding authors upon request.

## Electronic supplementary material

Supplementary Information

Description of Additional Supplementary Files

Supplementary Data 1

Supplementary Data 2

Supplementary Data 3
