# Supplementary material for: Distinct Campylobacter fetus lineages adapted as livestock pathogens and human pathobionts in the intestinal microbiota
Source: Nat Commun. 2017 Nov 8;8:1367. doi: 10.1038/s41467-017-01449-9 (PMC5678084; doi:10.1038/s41467-017-01449-9)
Supplement: Supplementary file 1 — Supplementary Information [file 41467_2017_1449_MOESM1_ESM.pdf]

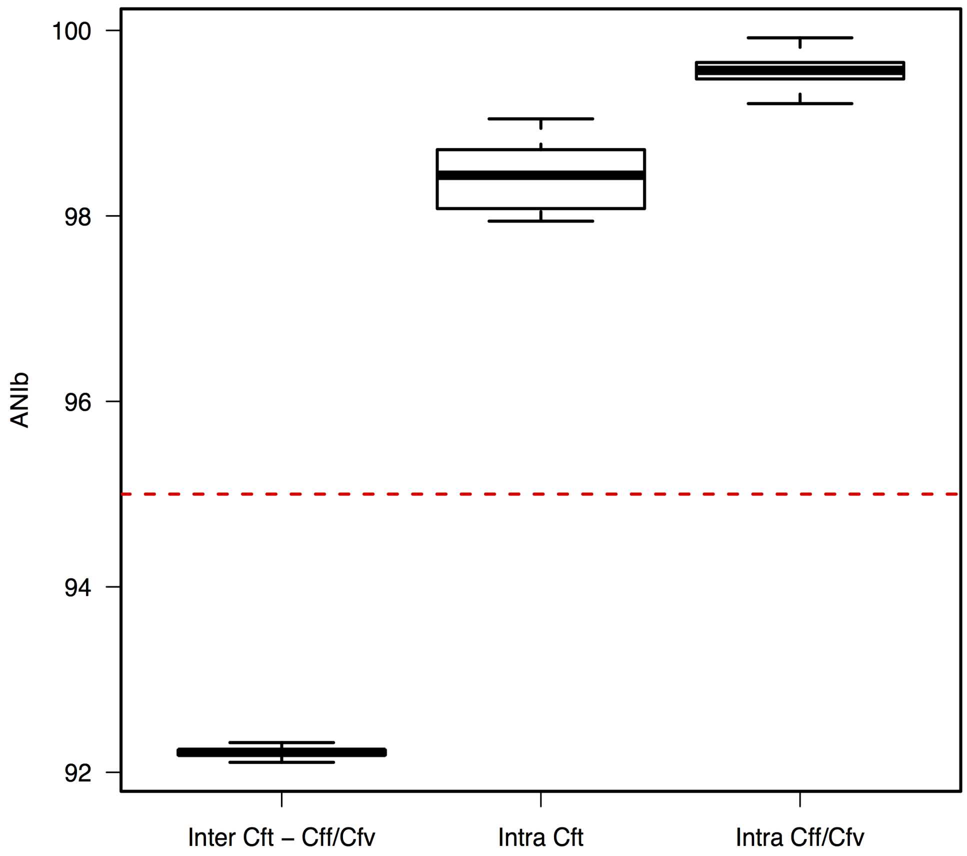

Supplementary Figure 1. Average Nucleotide Identity (ANI). Boxplots showing the ANI values. a) between Cft and Cff/Cfv, b) within Cft and c) within Cff/Cfv. The red dashed line indicates the accepted species threshold of ANI = 95%.

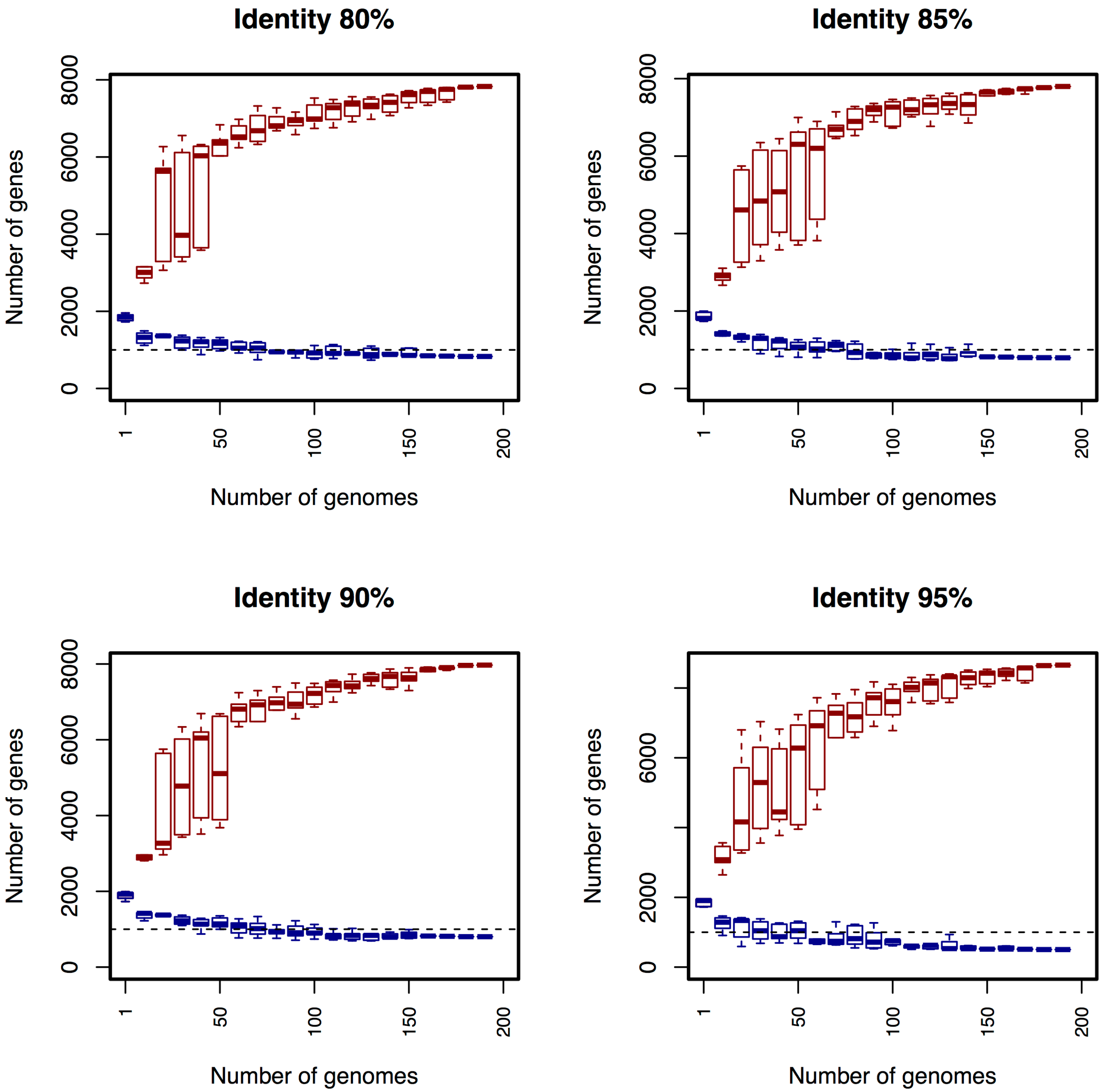

Supplementary Figure 2. Pan-genome plots. The size of the core and accessory genomes for the complete *C. fetus* dataset was inferred by varying the identity cut-off from 80% to 95%. Blue boxes indicate the core genome size variation and red boxes the accessory genome size variation for subsamples from 1 to 182 genomes. The horizontal dashed line indicates 1,000 genes.

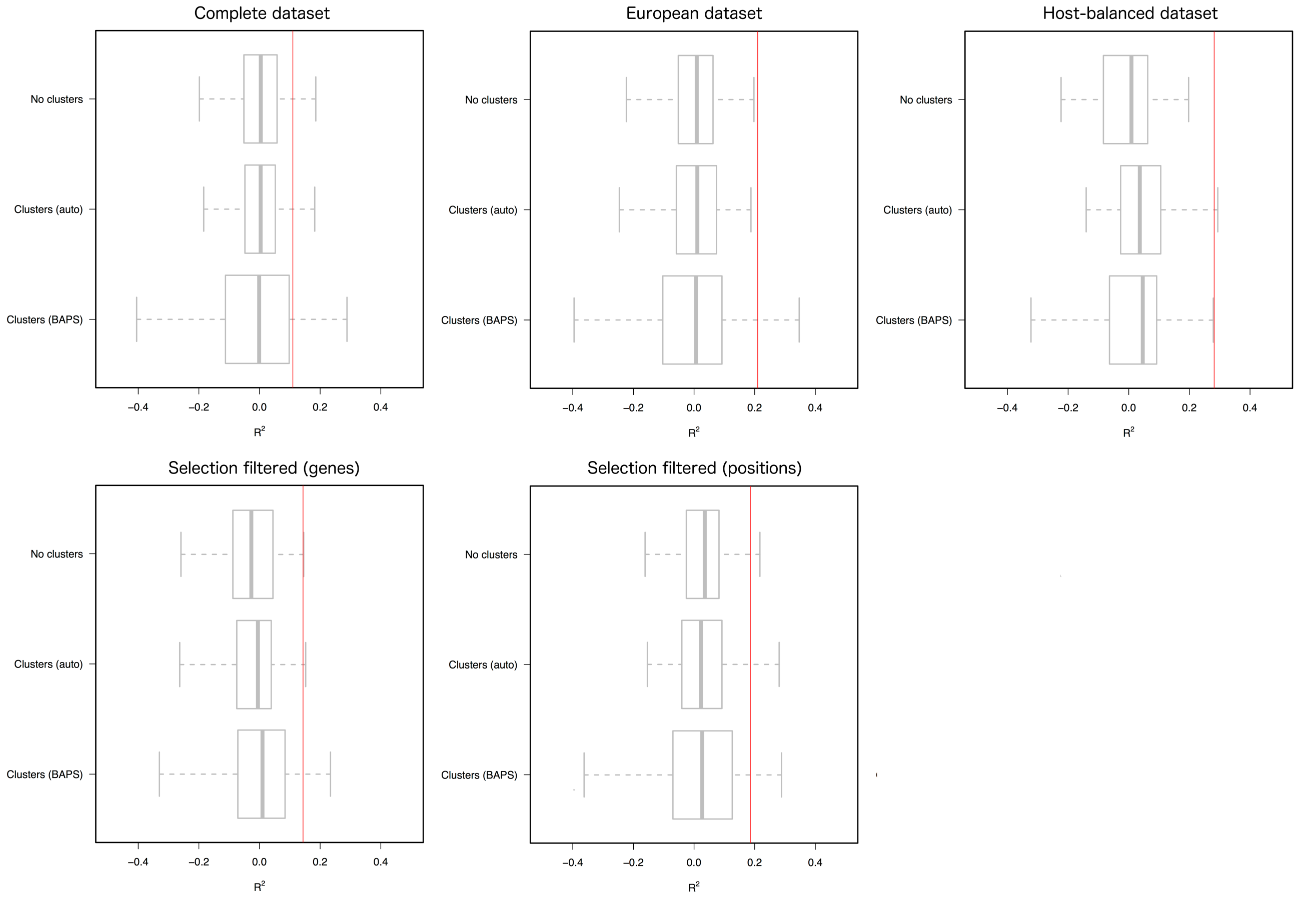

Supplementary Figure 3. Root-to-tip vs. isolation year regression analysis. Boxplots show the distribution of R^2 values (for the root-to-tip distance vs. year of isolation linear regression) obtained by making 1,000 random permutations of the isolation year. The analysis was performed with the complete dataset, a subsampled dataset just containing strains isolated in Europe, a subsampled dataset containing the same number of genomes from each host and two dataset in which sites under selection were removed from the alignment. Each one was analyzed with the standard random permutation approach, the clustered permutation approach and also a clustered permutation determined by the BAPS clusters. The real R^2 value for each dataset is shown as a vertical red line.

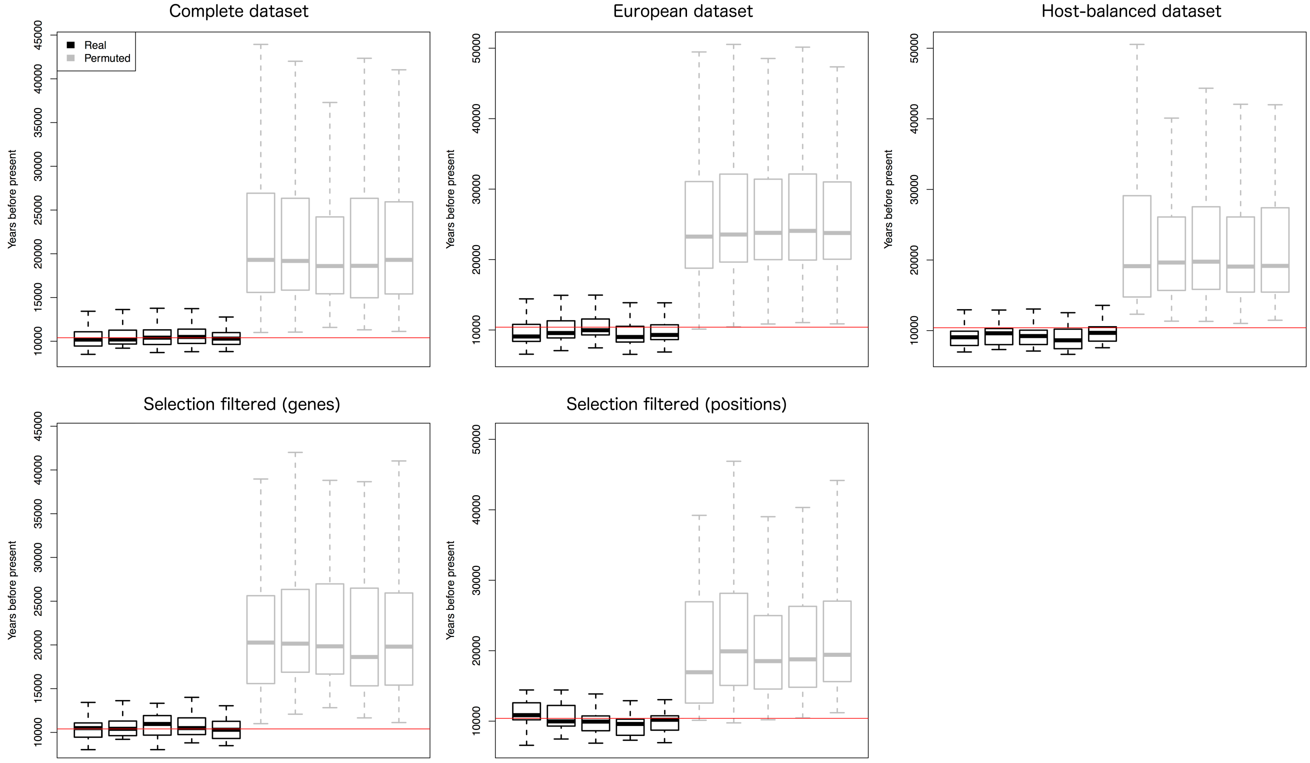

Supplementary Figure 4. Bayesian clustered permutation analysis. Boxplots show the distribution of the estimated time for the most recent common ancestor (tMRCA) of the *C. fetus* population obtained with the complete dataset, a subsampled dataset just containing strains isolated in Europe, a subsampled dataset containing the same number of genomes from each host and two dataset in which sites under selection were removed from the alignment. The black boxes represent the posterior distribution of tMRCA estimates using the real isolation year and the grey boxes represent the posterior distribution of tMRCA estimates obtained with the clustered permutation approach. The red horizontal line indicates the real tMRCA estimate.

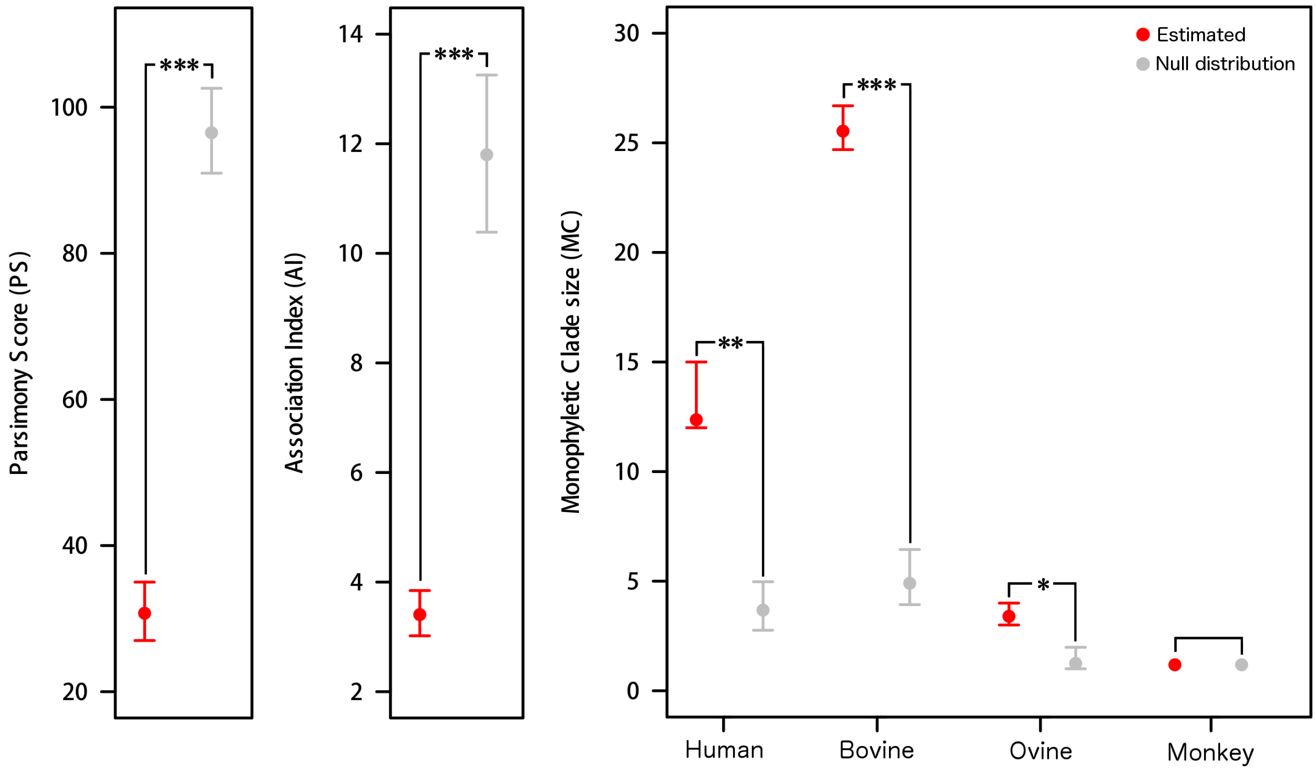

Supplementary Figure 5. Host-association analysis. This figure shows the results for the Bayesian-Tip Association Significance (BaTS) analysis performed to test the correlation between the population structure defined by the *C. fetus* phylogeny and the distribution of hosts. The estimated values of the Parsimony Score (PS), the Association Index (AI) and the Monophyletic Clade (MC) size are shown in red. The null distribution values for the same statistics were calculated by performing 1,000 bootstrap samples on the hosts, and are shown in grey.

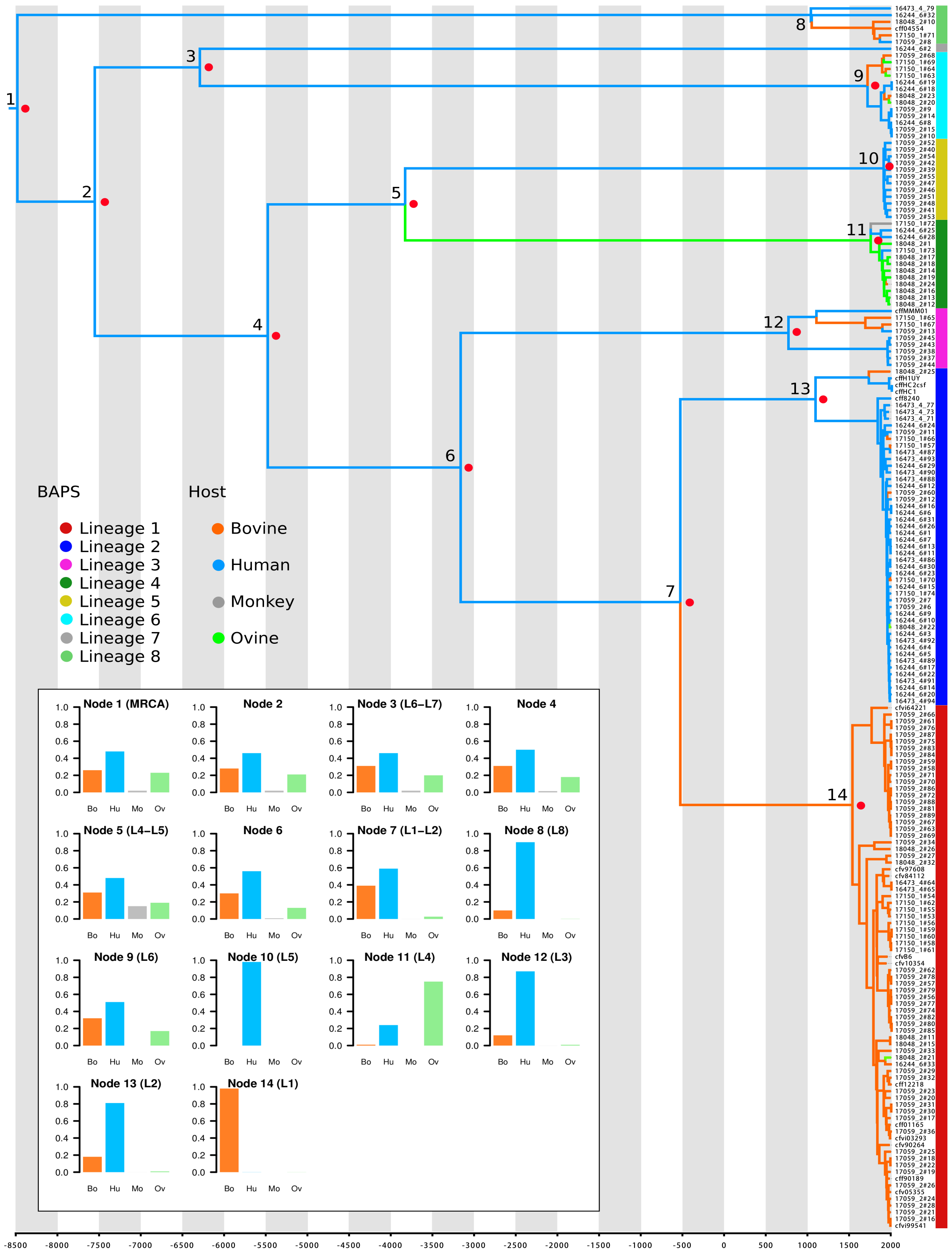

Supplementary Figure 6. Ancestral host reconstruction. The ancestral host reconstructions are presented as colored internal branches of the three according to the most probable host. The rightmost color strip indicates the BAPS clusters. The barplots show the posterior probability for each host in the ancestral nodes that are numbered in the phylogeny. Red dots indicates nodes with high posterior probability (>0.95).

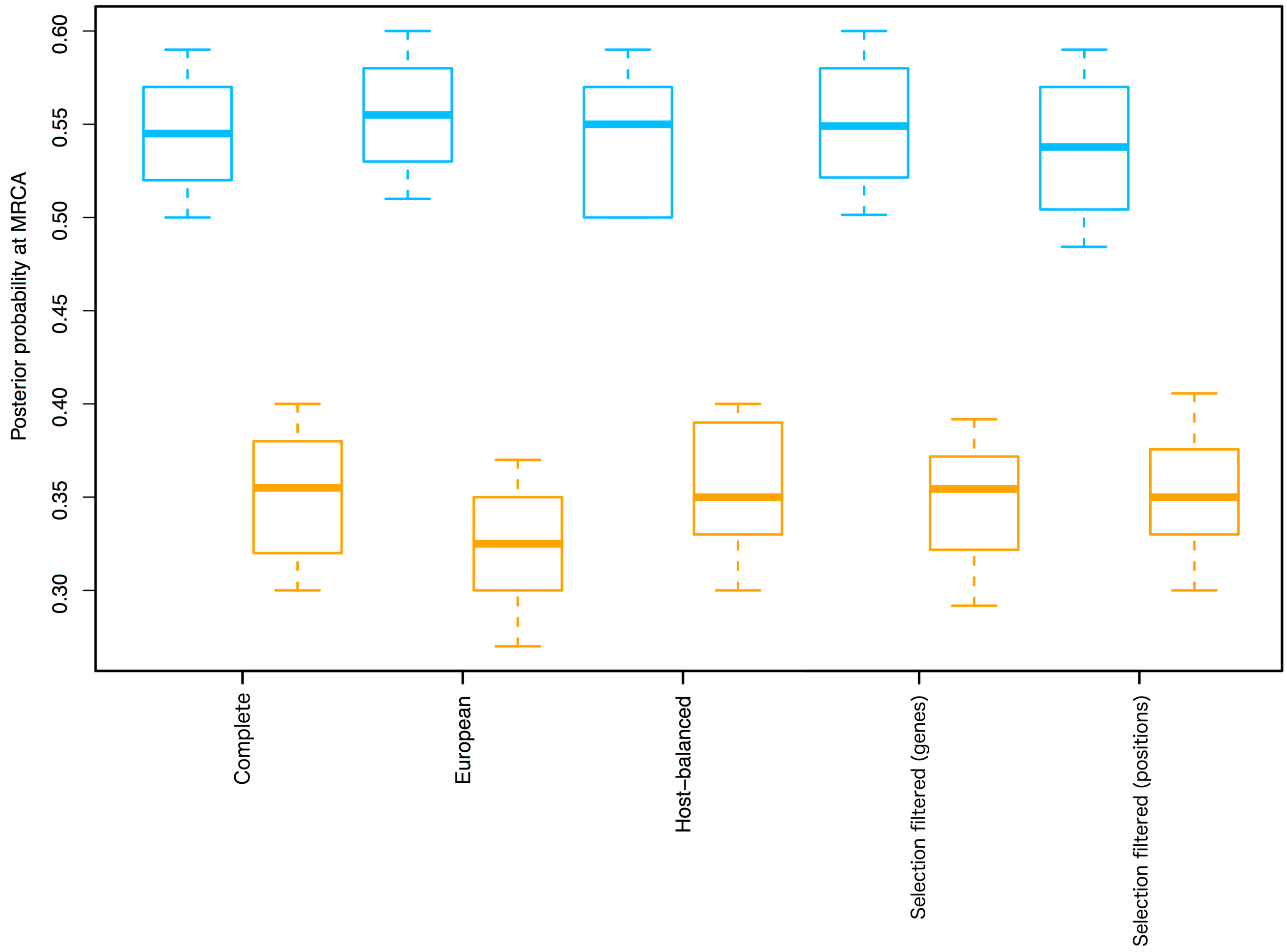

Supplementary Figure 7. Host ancestral state at the most recent common ancestor. Boxes show the estimations for the bovine (orange) or human (blue) host ancestral states at *C. fetus* most recent common ancestor (MRCA). Independent runs were performed using the complete dataset, a subsampled dataset just containing strains isolated in Europe, a subsampled dataset containing the same number of genomes from each host and two dataset in which sites under selection were removed from the alignment.

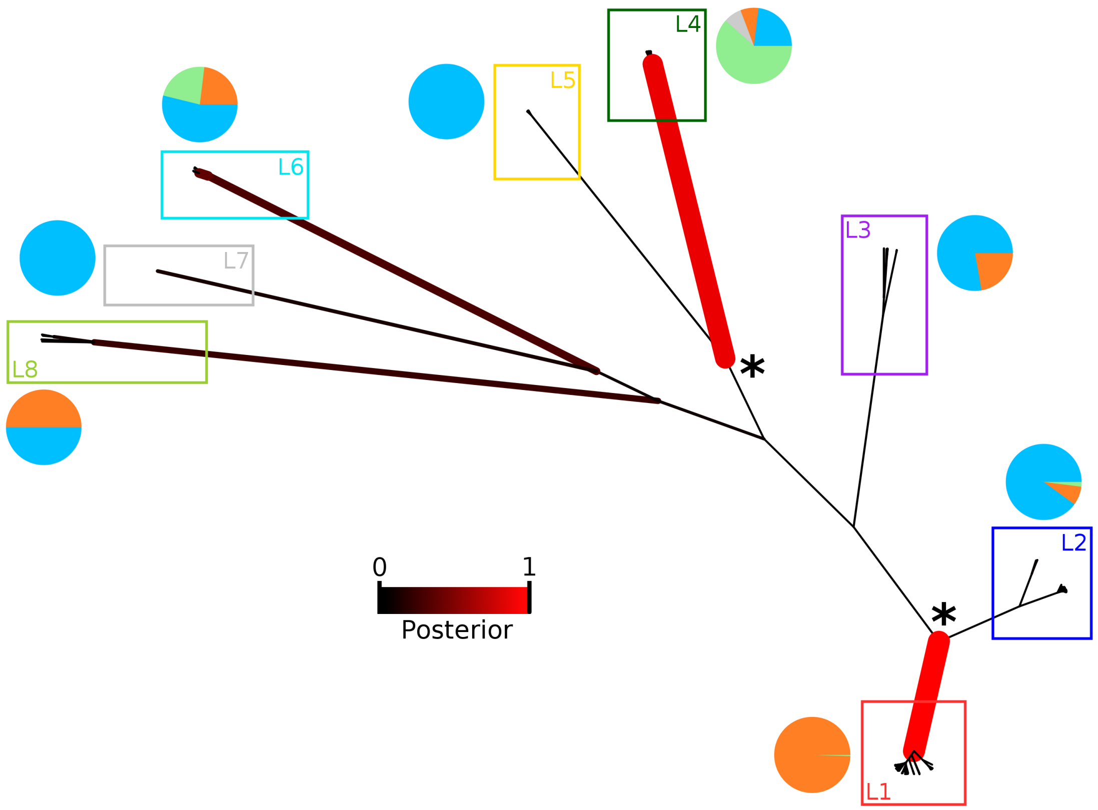

Supplementary Figure 8. Hosts distribution across lineages. The analysis implemented in the software TreeBreaker shows branches in the core genome phylogeny where the distribution of hosts significantly changed. The redness and thickness of the branches are proportional to the posterior probability for a significant change in the host distribution. Pie charts indicate the actual host distribution at each BAPS cluster (identified as colored squares). The asterisks indicate branches with posterior probability > 0.95.

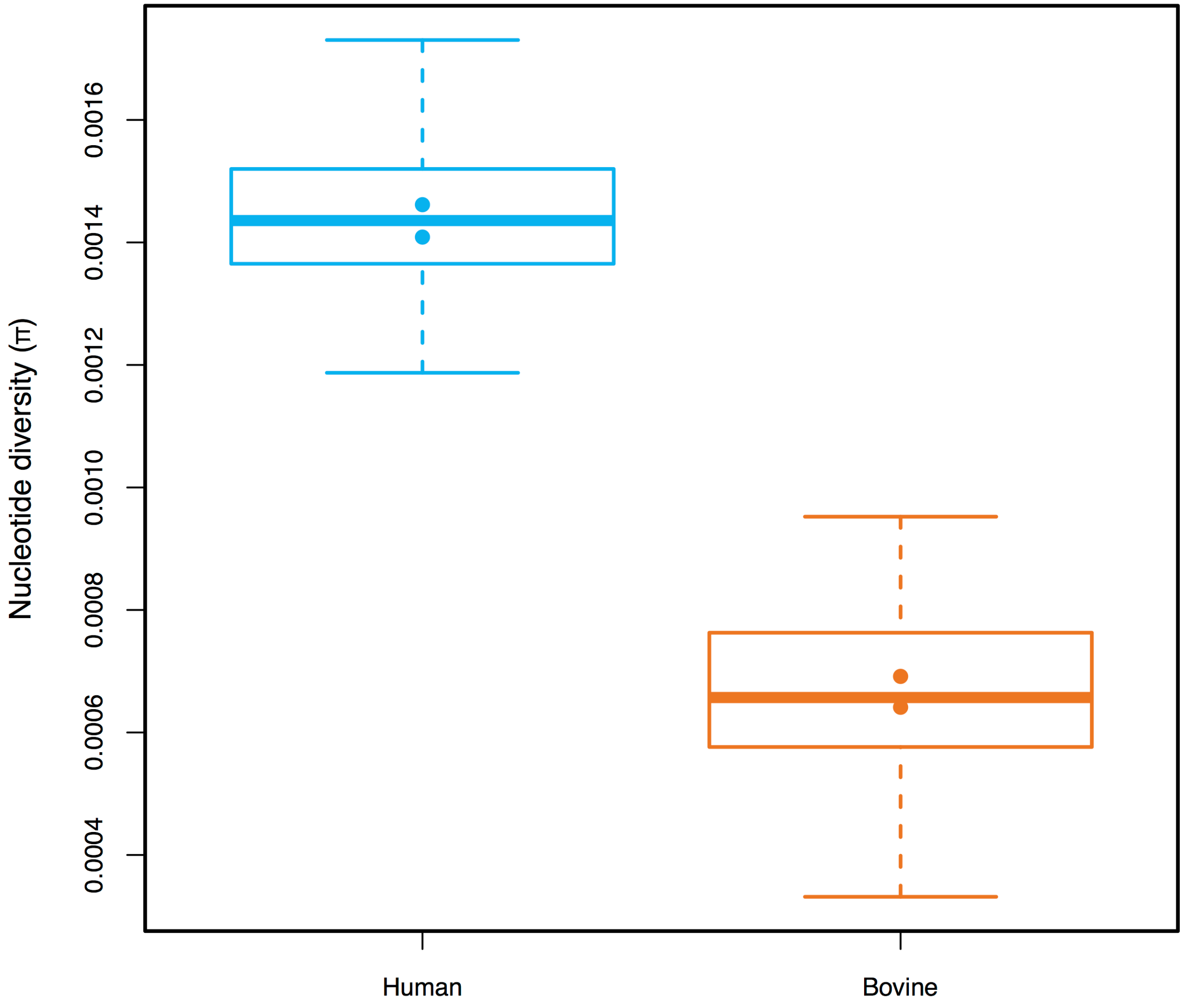

Supplementary Figure 9. Nucleotide diversity among host types. Boxplots show the distribution of sequence nucleotide diversity (Π) calculated between pairs of genomes belonging to the bovine or human host type.

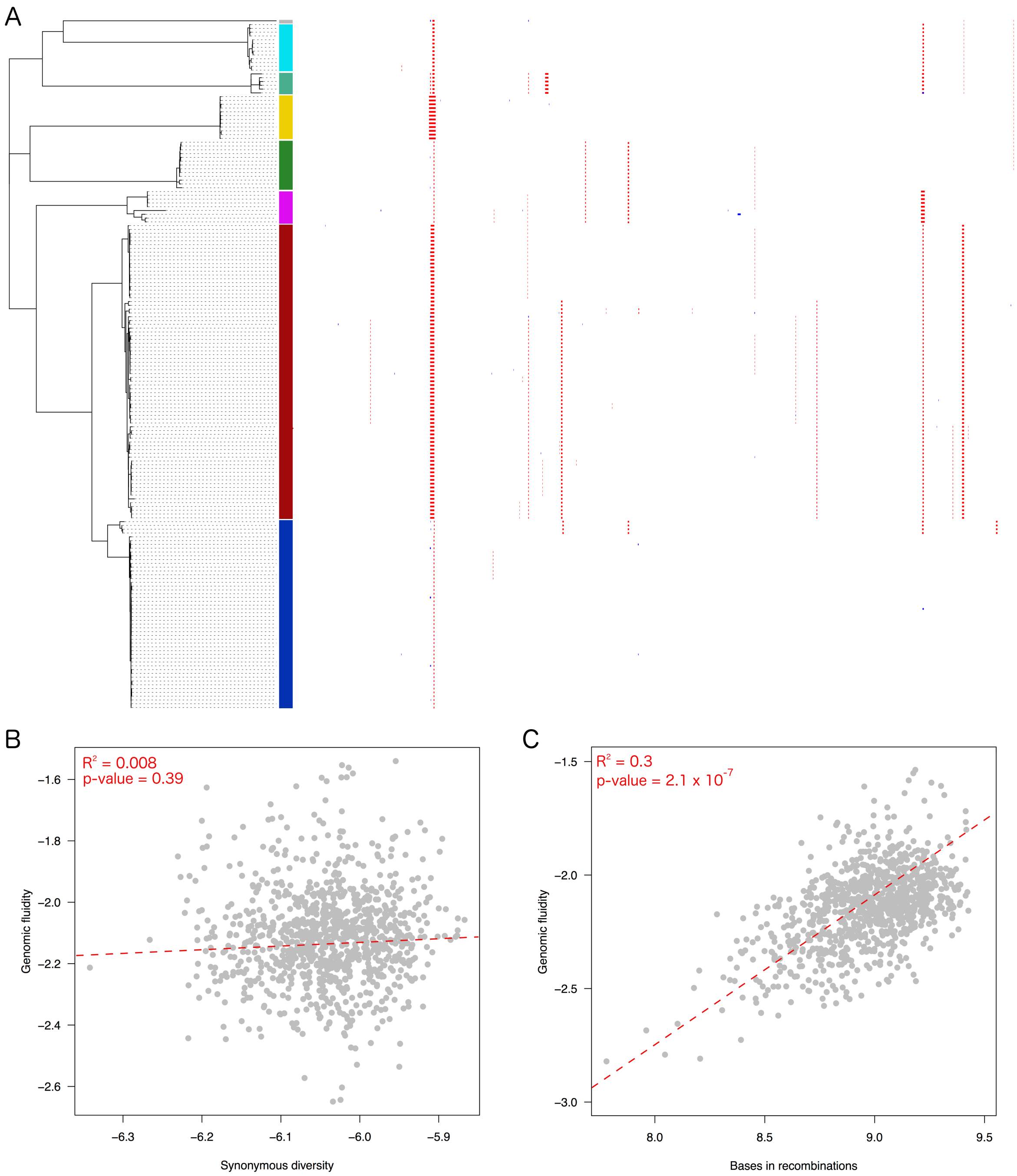

Supplementary Figure 10. Signals of adaptive evolution. a) Recombination analysis using Gubbins. The tree was built from non-recombinant regions in the core genome alignment. The colored strip highlights the eight BAPS clusters. Red blocks indicate recombinant regions along the core genome. The presence of shared recombinant blocks in divergent lineages (different BAPS clusters) is an evidence of convergent evolution. b) Linear regression analysis of genomic fluidity (accessory genome) against synonym diversity (core genome). The absence of a significant correlation between these two measures is against the presence of neutral evolution. c) Linear regression of genomic fluidity the number of recombinant bases. The positive significant correlation supports adaptive rather than neutral evolution.

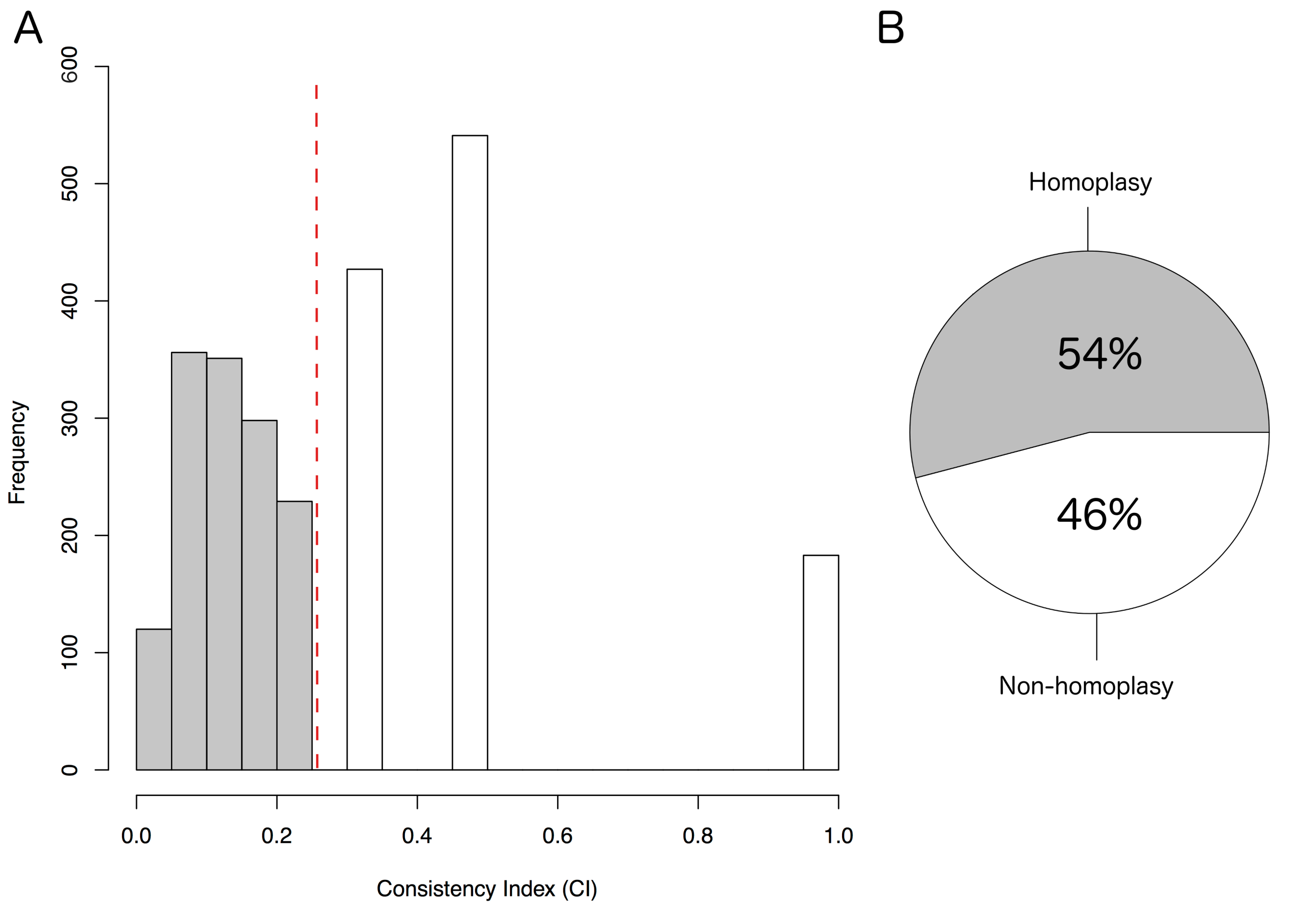

Supplementary Figure 11. Homoplasy in the accessory genome. a) Histogram of consistency index (CI) values obtained from each accessory gene. The red dashed line indicates the empirical threshold (CI < 0.25) set for considering a gene to have homoplasy (grey bars). b) Pie chart showing the proportion of accessory genes with (54%) and without (46%) homoplasy according to CI < 0.25.

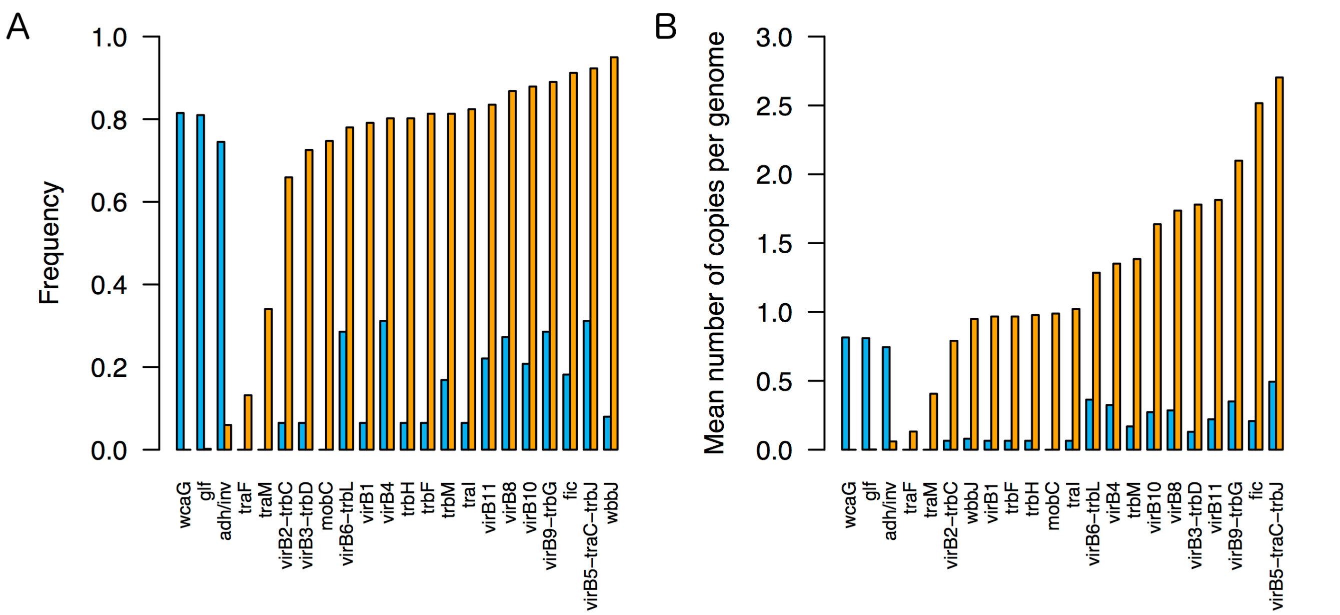

Supplementary Figure 12. Abundance of accessory genes (virulence factors). Bar plots showing a) the frequency or b) the mean number of copies per genome for each virulence accessory gene presented in presented in Figure 3a. For calculating the frequency, gene abundances (number of copies) were transformed into presence/absence (1/0). Bar colors represent the bovine (orange) and human (blue) lineages.

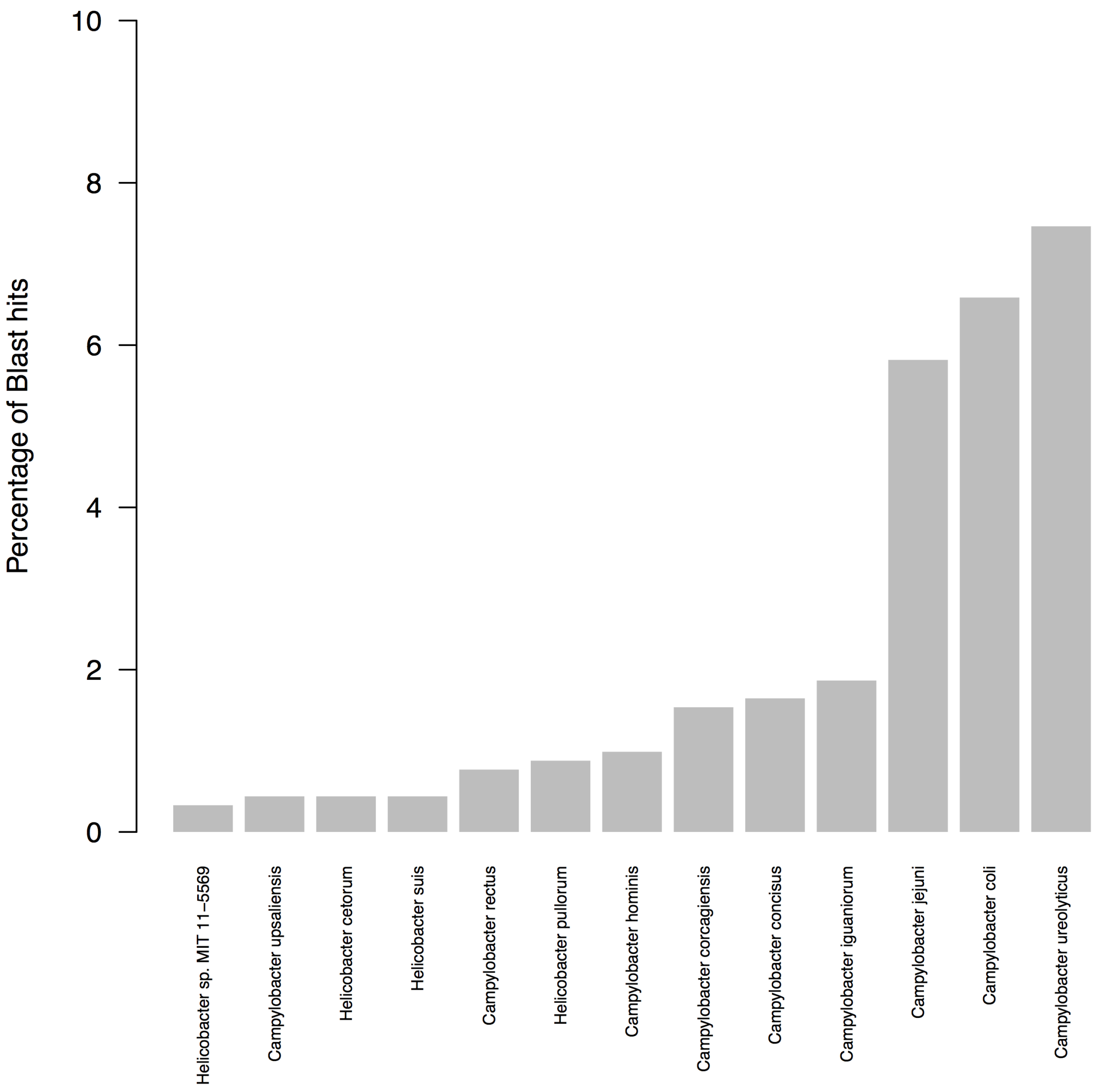

Supplementary Figure 13. Potential origin of horizontally-acquired virulence genes. The set of accessory genes identified as virulence factors that are known to be highly frequent inside mobile elements like pathogenicity islands and plasmids, were compared with the NCBI nr database in order to identify non-*C. fetus* homologs present in related species that could suggest their origin. Bars represent the percentage of top hits against most frequent species.

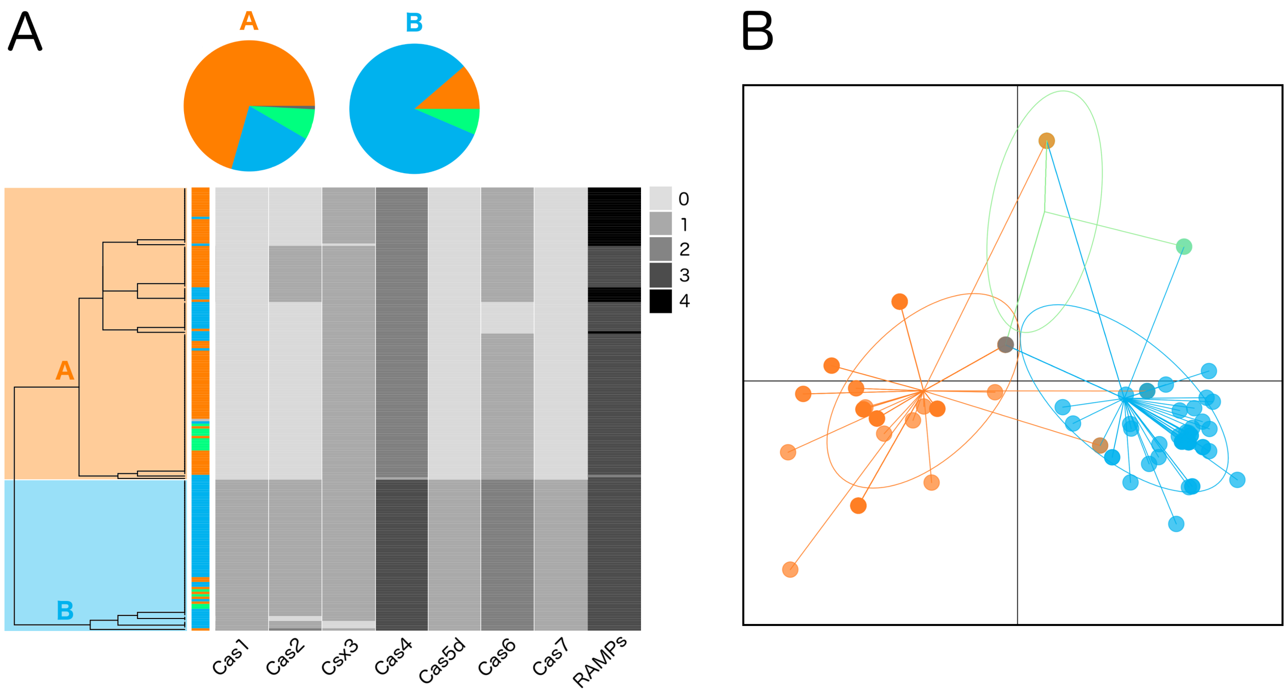

Supplementary Figure 14. CRISPR/Cas analysis. a) Heatmap based on the abundance patterns of Cas protein families in *C. fetus* genomes, the hierarchical clustering shows two groups enriched in bovine and non-bovine strains. b) Discriminant Analysis of Principal Components (DAPC) based on abundance patterns of CRISPR spacers showing a great discriminatory power between hosts.

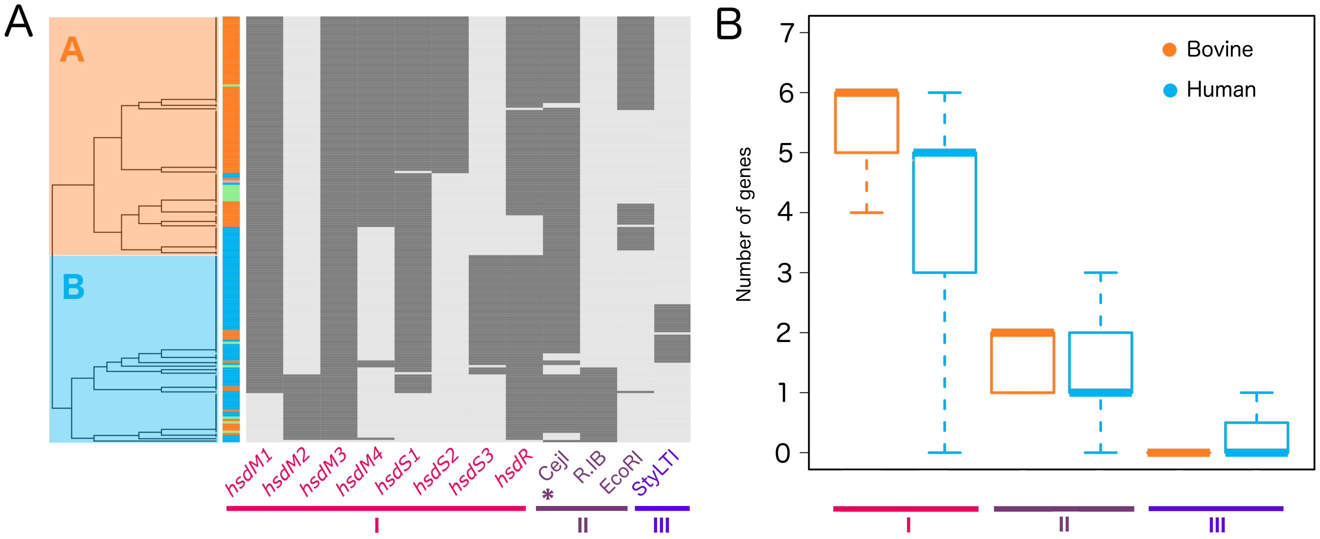

Supplementary Figure 15. Restriction-modification systems. a) Heatmap based on the abundance patterns of type I, II and III Restriction-Modification genes, the hierarchical clustering shows two groups enriched in bovine and human strains. b) Boxplots showing the abundance of different types of Restriction-Modification genes in bovine and human lineages.
