# Supplementary material for: Distinct Campylobacter fetus lineages adapted as livestock pathogens and human pathobionts in the intestinal microbiota
Source: Nat Commun. 2017 Nov 8;8:1367. doi: 10.1038/s41467-017-01449-9 (PMC5678084; doi:10.1038/s41467-017-01449-9)
Supplement: Supplementary file 2 — Description of Additional Supplementary Files [file 41467_2017_1449_MOESM2_ESM.pdf]

Description of Additional Supplementary Files

File Name: Supplementary Data 1
Description: Metadata associated to each genome used in this study, including those sequenced here and publicly available.

File Name: Supplementary Data 2
Description: Genes identified to be evolving under positive selection.

File Name: Supplementary Data 3
Description: Identity of virulence factors against the NCBI nr database.
